# Supplementary material for: The Impact of Sciatic Nerve Injury on Extracellular Matrix of Lower Limb Muscle and Thoracolumbar Fascia: An Observational Study
Source: Int J Mol Sci. 2024 Aug 16;25(16):8945. doi: 10.3390/ijms25168945 (PMC11354760; doi:10.3390/ijms25168945)
Supplement: Supplementary file 1 [file ijms-25-08945-s001.zip › ijms-3112456-supplementary.pdf]

Supplementary Materials

The Impact of Sciatic Nerve Injury on Extracellular Matrix of Lower Limb Muscle and Thoracolumbar Fascia: An Observational Study

Xiaoxiao Zhao, Caterina Fede, Lucia Petrelli, Carmelo Pirri, Elena Stocco, Chenglei Fan, Andrea Porzionato, Raffaele De Caro, Stefano Masiero and Carla Stecco

Supplementary Figure S1. Representative image of Sprague-Dawley rats' Footprints after 6 weeks from surgery.

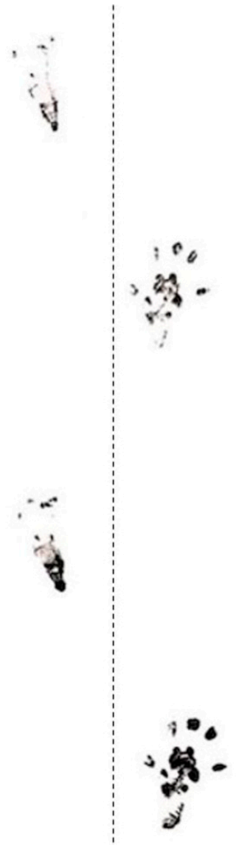

S1. The left footprint corresponds to the injured side whereas the right footprint shows the paw imprint of contralateral side.

Supplementary Table S1. Weight (kg) of Rats in week 1, 3, and 6

Rat No.	Weight (kg)		
	week1	week3	week6
SD1	0.61	0.55	0.56
SD2	0.63	0.54	0.56
SD3	0.64	0.56	0.54
SD4	0.60	0.54	0.60
SD5	0.58	0.58	0.56
SD6	0.59	0.56	0.57
SD7	0.53	0.56	0.57
SD8	0.59	0.59	0.58
SD9	0.57	0.57	0.56
SD10	0.57	0.62	0.59
SD11	0.56	0.64	0.61
SD12	0.55	0.63	0.61
MEAN	0.585	0.578	0.576
SD	0.0321	0.0346	0.0224

Supplementary Figure S2. Image analysis of the area percentage of collagen content of gastrocnemius muscle

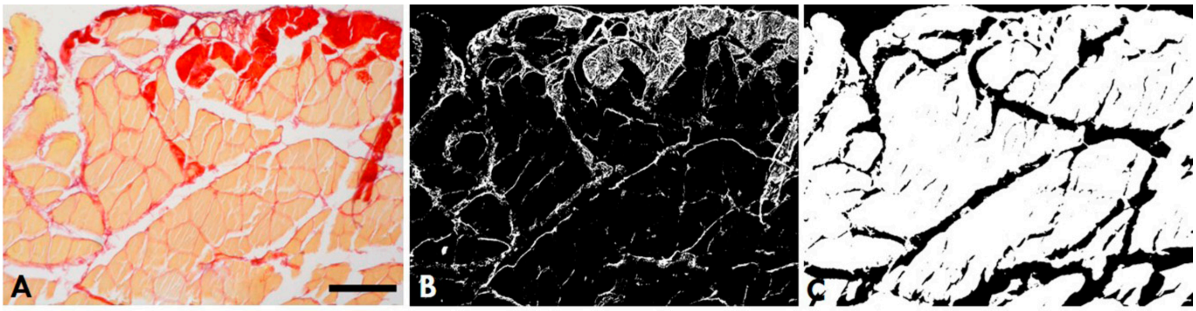

S2. Images processing and morphometric analysis: A: Representative original image stained by Picrosirius Red acquired by Leica DMR microscope (Scale Bars 150 μm); B: Binary images segmented by ImageJ software; C: Second binary images measured by ImageJ software for area percentage of collagen content of gastrocnemius muscle.
